# A Twin Protection Effect? Explaining Twin Survival Advantages with a Two-Process Mortality Model

**DOI:** 10.1371/journal.pone.0154774

**Published:** 2016-05-18

**Authors:** David J. Sharrow, James J. Anderson

**Affiliations:** University of Washington, Seattle, Washington, United States of America; University of Palermo, ITALY

## Abstract

Twin studies that focus on the correlation in age-at-death between twin pairs have yielded important insights into the heritability and role of genetic factors in determining lifespan, but less attention is paid to the biological and social role of zygosity itself in determining survival across the entire life course. Using data from the Danish Twin Registry and the Human Mortality Database, we show that monozygotic twins have greater cumulative survival proportions at nearly every age compared to dizygotic twins and the Danish general population. We examine this survival advantage by fitting these data with a two-process mortality model that partitions survivorship patterns into extrinsic and intrinsic mortality processes roughly corresponding to acute, environmental and chronic, biological origins. We find intrinsic processes confer a survival advantage at older ages for males, while at younger ages, all monozygotic twins show a health protection effect against extrinsic death akin to a marriage protection effect. While existing research suggests an increasingly important role for genetic factors at very advanced ages, we conclude that the social closeness of monozygotic twins is a plausible driver of the survival advantage at ages <65.

## Introduction

Due to the social and economic consequences of variation in human lifespan there is considerable interest in identifying the extent of social, environmental, and biological determinants of survival patterns in humans. While studies of extreme longevity clustered within human families have indicated at least some genetic role in determining lifespan at very advanced ages [1–4], twin studies, which offer the opportunity to disentangle the genetic and environmental factors for a given trait, indicate genetic factors are responsible for only a modest amount of the variation (20–30%) in human lifespan [5–7] and that the role of genetic factors is minimal before age 60, but increases thereafter [4, 8]. Although twin studies that focus on the correlation in age-at-death have yielded important insights into the role of genetics in human lifespan, the determinants of human survival patterns are immensely complex and change with age—i.e. while genetic factors play an increasingly larger role at advanced ages, environmental, social, and behavioral factors influence survival patterns much more heavily at younger ages. Perhaps owing to this complexity and the traditional structure of twin survival studies, less is known about differences in survival across age by zygosity, the underlying mortality processes that produce these differences, or the role of zygosity itself in shaping age patterns of survival.

**Fig 1:**
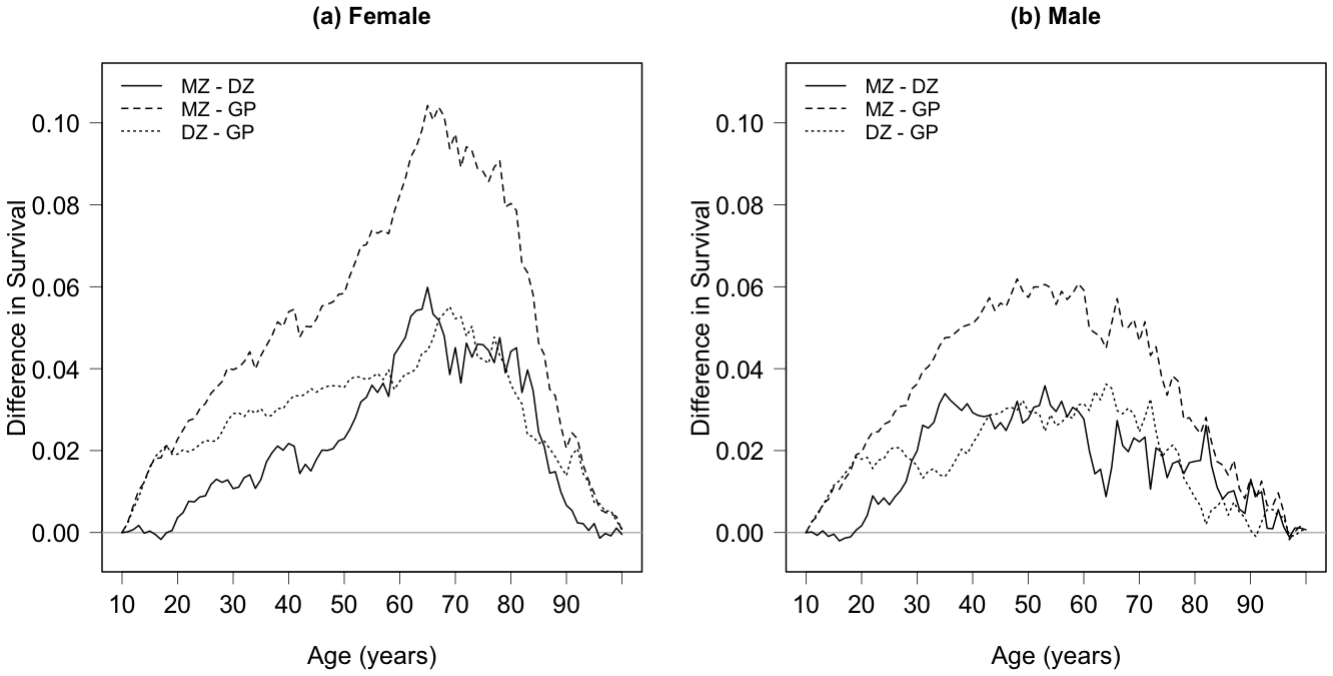
Pairwise differences in cumulative survival proportion among Danish twins and the general population. Difference in cumulative survival proportion (l_x_ column of the life table) between MZ and like-sex DZ twins born in Denmark 1870–1900 (solid line), MZ twins and the Danish general population cohort 1870–1900 (dashed line), and DZ twins and the Danish general population cohort 1870–1900 (dotted line). GP: general population, DZ: dizygotic, MZ: monozygotic.

Fig 1 shows the difference in the cumulative survival proportion by age (*l*_*x*_ column of the life table) between all monozygotic (MZ) and like-sex dizygotic (DZ) twins born in Denmark from 1870–1900 and the 1870–1900 Danish general population cohort. MZ twins of both sexes have a survival advantage at nearly every age over their DZ counterparts and both zygositites enjoy a survival advantage over the general population cohort from the same time period. Our focus is the origins of these twin advantages in survival across age.

We aim to quantify the extrinsic and intrinsic contributions to survival across age and by zygosity by fitting a two-process vitality model to survival data for the twin and general population cohorts shown in Fig 1. In the vitality framework, mortality is partitioned into either an intrinsic process where death is the result of the cumulative and incremental degradation of survival capacity with age or an extrinsic process in which death results from an acute environmental challenge. While the extrinsic process is unlikely to be closely associated with genetic endowment, the incremental decline in survival capacity—i.e. vitality—mimics the process of senescence and is therefore postulated to be influenced by life-long cumulative health behaviors and, to a certain extent, genetic inheritance. Note that these two processes primarily influence mortality at different ages—extrinsic in adulthood and intrinsic in old age—and that the distinction of the two processes ultimately relies on the time scale to death (extrinsic is short-term and acute; intrinsic is long-term and chronic) rather than on cause of death per se. That said we do not wish to convey that we are directly quantifying the environmental and genetic contributions to longevity as is done in conventional genomic studies using twin data. While this quantification is indeed an important task, it has been explored in numerous other published works [4–6, 9–11] and our model framework is not designed to directly make this distinction. However, the vitality model’s decomposition of the overall survival function into plausible underlying mortality processes that shape the survivorship patterns among twins and the general population can reveal important insights into the factors that drive survival across the life course in general. Furthermore, these partitions should link to a number of direct causes that have implications for programs aimed at improving quality of life and meeting the demands of ever increasing human lifespans.

## Materials and Methods

### Data

The Danish Twin Registry (DTR), a national register administered in accordance with the Processing of Personal Data Act (Denmark), provided data for this study. The DTR Scientific Board approved this project and the dataset provided by the DTR for analysis in this study is anonymized.

The twins dataset consists of all monozygotic and like-sex dizygotic twin pairs born in Denmark from 1870–1900 [12, 13]. To identify all twin births, birth registrars from all parishes within Denmark were manually reviewed and zygosity was established through a questionnaire sent to the surviving twin(s) or closest surviving relative. It was not possible to follow up with twins who died or emigrated at a young age, so pairs where one twin died or emigrated before age six are excluded by the DTR. Because there is no childhood mortality in this dataset and due to the modeling framework described in the next section, we further truncate the data to twin pairs where both survived to age 10. Table 1 shows the total number of individuals by zygosity and age of survival for both sexes. This dataset consists of 2,958 like-sex twin pairs total and 2,932 like-sex twin pairs where both survived to age 10. All twins born in the 1870–1900 cohort are now deceased and thus their length of life is known. The validity of twin zygosity classification by this manner has shown to be of high accuracy [8, 14, 15] and studies suggest twin survival is a good model for studying longevity [16].

**Table 1:** Total number of individuals by zygosity and age to which both co-twins survive. MZ: monozygotic, DZ: dizygotic.

	All (6+)	10+
MZ	2124	2104
DZ	3792	3760
Total:	5916	5864

Survival data for the 1870–1900 Danish general population cohorts consisting of all births from 1870–1900 in Denmark were obtained from the Human Mortality Database [17] and are also truncated at age 10 for model fitting.

### Two-Process Mortality Model

**Fig 2:**
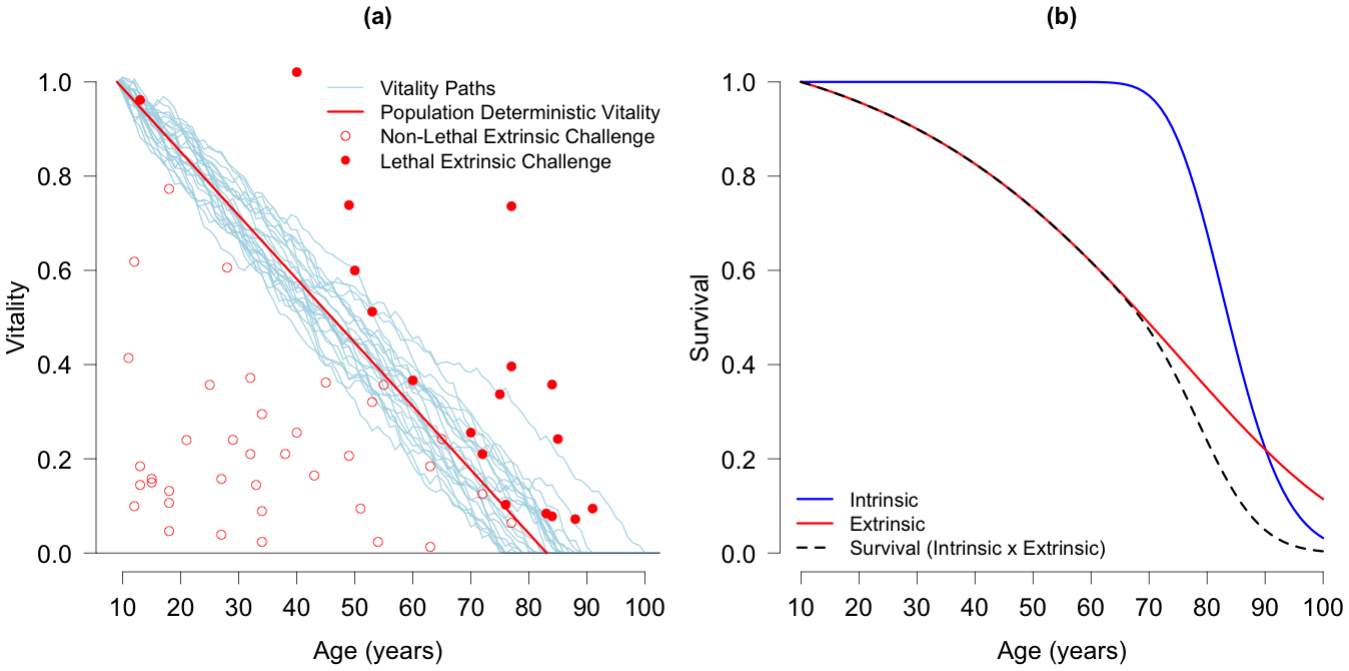
Two-process Vitality Model Illustrated. (a) The intersection of individual stochastically declining vitality paths (light blue lines) with the zero boundary marks intrinsic death. Extrinsic death occurs when a challenge (filled, red circles) exceeds the deterministic population vitality (red line). (b) shows the two-process vitality model in terms of age-specific survival proportions. Intrinsic survival (blue line) remains at 1 until later ages and extrinsic challenges dominate mortality in early life as shown with the extrinsic survival function (red line). The total survival curve is shown with a dashed, black line and is the product of the intrinsic and extrinsic survival functions as shown in Eq 1. Both figures produced with parameter values: r = 0.0135; s = 0.0126; λ = 0.0449; β = 0.3999.

We fit the twin and general population data with a four-parameter version of a two-process vitality model [18] that has been used to describe varying demographic phenomenon including the historical pattern of Swedish mortality [18], late-middle life/early-old age mortality accelerations [19], and the breakdown of the log-linear relationship of Gompertz mortality parameters in the 20^th^ century [20]. Fig 2a shows how the two mortality processes work over age. The blue lines depict the intrinsic process where an individual’s death occurs with the passage of ‘vitality,’ an abstract measure of survival capacity, by a hidden Markov process to an absorbing boundary representing death [18, 21–23]. Because the intrinsic process is a rudimentary representation of the individual physiological processes leading to senescence, it has been described as a “process point-of-view” approach [24]. The red plotting symbols in Fig 2a depict the extrinsic process, which is equivalent to the Strehler and Mildvan [25] interpretation of Gompertz-type models [26–29]. Acute environmental challenges that instantaneously exhaust remaining vitality result in extrinsic death and are represented by the filled red circles in Fig 2a. The vitality framework merges Gompertz-type force of mortality models with the “process point-of-view” approach and shifts the focus from explaining how the “force of mortality” (i.e. the mortality rate) changes with age to how the underlying mortality processes change with age.

### Estimating Model Parameters

The model is fit to the cumulative age pattern of survival for a population (excluding childhood mortality), which is governed by a total of four parameters: *r*, the mean rate of vitality loss and *s*, the variability in the rate of vitality loss, control intrinsic mortality, while λ, the frequency of extrinsic challenges, and β, the mean magnitude of extrinsic challenges (exponentially distributed), control extrinsic mortality. Panel b of Fig 2 depicts the vitality model in terms of the age-specific cumulative survival curves produced by the intrinsic and extrinsic processes. The mathematical development of the two mortality processes is described elsewhere [18, 21–23]. Here we show the equations defining the intrinsic and extrinsic components of the survival curve.

The total cumulative age pattern of survival is the product of the extrinsic and intrinsic survival curves as shown in Eq 1.

$$l_{x}=l_{x}^{i}\times l_{x}^{e}$$ (1)

*l*_*x*_ is the total survival curve and $l_{x}^{e}$ and $l_{x}^{i}$ are the extrinsic and intrinsic survival curves respectively. Intrinsic survival, shown with the solid, blue line in Fig 2b, is (from Li and Anderson [18])

$$l_{x}^{i}=\Phi \left(\frac{1-rx}{s\sqrt{x}}\right)-\text{exp}\left(\frac{2r}{s^{2}}\right)\Phi \left(-\frac{1+rx}{s\sqrt{x}}\right)$$ (2)

where Φ is a cumulative normal distribution and *x* is age. Extrinsic survival (solid, red line in Fig 2b), $l_{x}^{e}$ is

$$l_{x}^{e}=\text{exp}\left(-\frac{\beta }{r}\times \frac{\lambda \text{exp}\left(-1/\beta \right)}{\text{exp}\left(rx/\beta \right)-1}\right)$$ (3)

Due to the interaction of the intrinsic and extrinsic processes (i.e. the extrinsic process is dependent on the distribution of vitality at each age, which is controlled by the two intrinsic parameters), the two-process approach cannot be expressed in a closed form that could be fit to data to yield parameter estimates. Li and Anderson [18] developed an approximate analytical solution that can be fit to data by assuming that vitality for the extrinsic process is a linear deterministic function, thus giving independent intrinsic and extrinsic parts. This closed form solution is the model we fit in this paper, however this solution does give slightly biased parameter estimates—somewhat low for *r* and β and somewhat high for *s* and λ. Li and Anderson provide a set of bias-correction formulas based on simulation with a numerical form of the model with greater interaction between the two processes [18]. Because the closed form version of the vitality model we fit in this paper has limited parameter interactions, we use a maximum likelihood method to estimate the model parameters for age interval survival data [30]. See Li and Anderson [18] and S1 Appendix for further details on model development and bias correction.

All model fitting is performed with the fitting routines provided in the ‘vitality’ package available on the Comprehensive R Archive Network (CRAN) (http://cran.r-project.org/web/packages/vitality/index.html). Once we’ve obtained parameter estimates and calculated the fitted survival curves from intrinsic and extrinsic processes using Eqs 2 and 3, we compare these quantities for the twins and general population.

### Intrinsic and Extrinsic Components from cohort data

A final note on interpretation concerns period versus cohort data. Because they are of biological origin, intrinsic parameters estimated from cohort data are by definition constant within the cohort and are expected to change slowly across cohorts. In contrast, extrinsic parameters, which reflect environmental conditions, may be considered constant in a given period but change from one period to the next [18]. Therefore, extrinsic parameters estimated from cohort data represent weighted averages of environmental conditions comprising the cohort.

## Results

**Fig 3:**
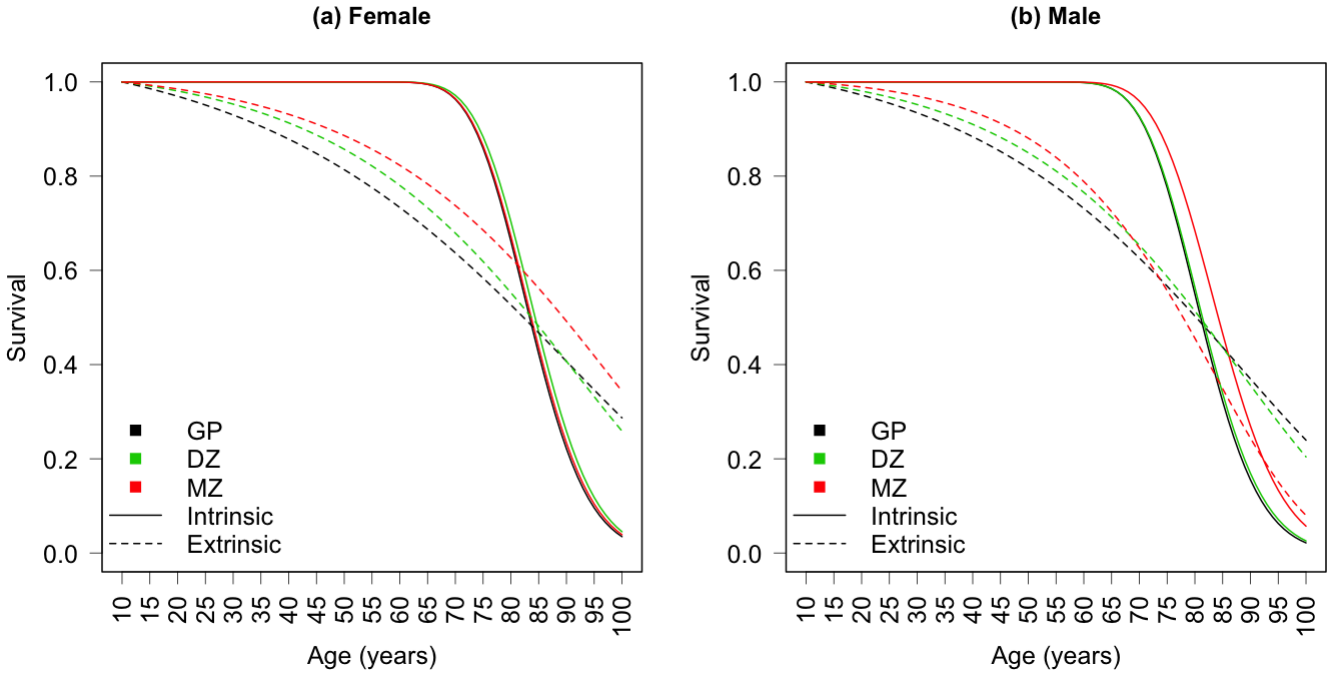
Fitted intrinsic and extrinsic age-specific survival functions. Fitted intrinsic (solid lines) and extrinsic (dashed lines) age-specific survival by sex and zygosity for the 1870–1900 Danish twin cohorts and the 1870–1900 Danish general population cohort. GP: general population, DZ: dizygotic, MZ: monozygotic.

**Fig 4:**
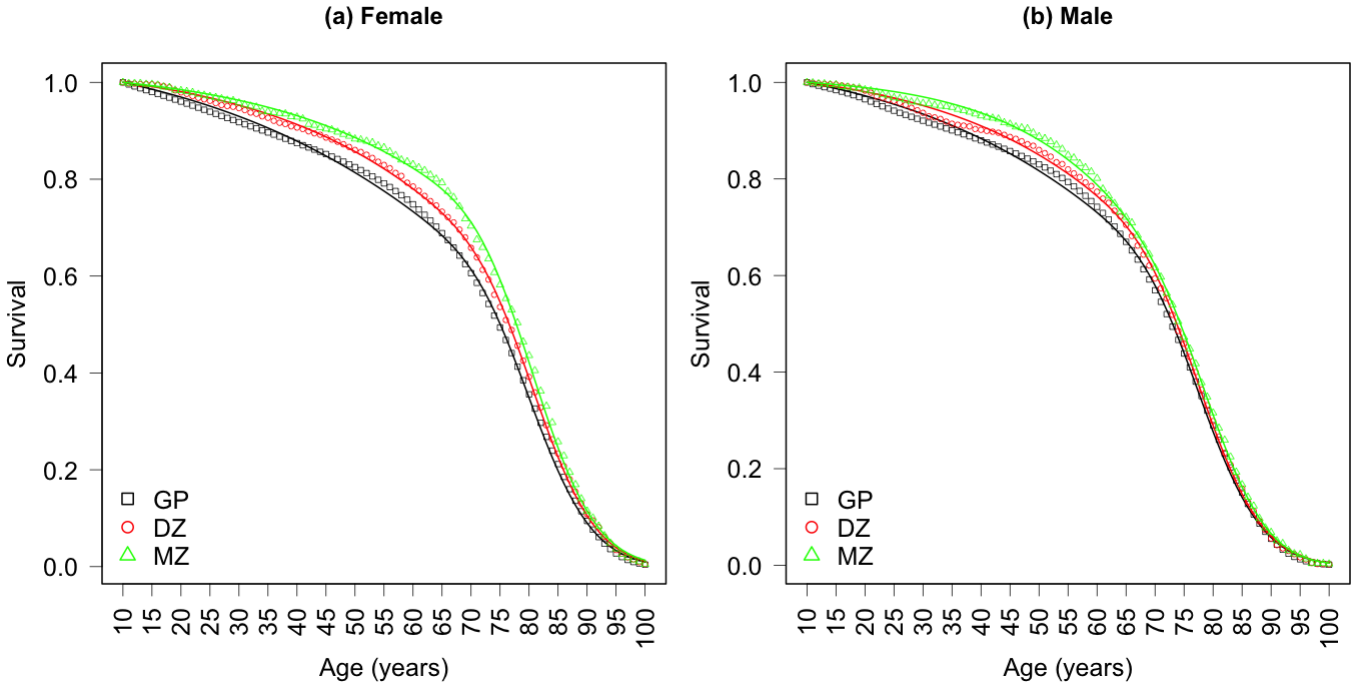
Cumulative survival data and model fit. Cumualtive survival data (plotting symbols) by zygosity and sex for the 1870–1900 Danish twin cohorts and the 1870–1900 Danish general population cohort. Smooth line through each set of survival data is the model fit. GP: general population, DZ: dizygotic, MZ: monozygotic.

Let us begin with a discussion of the cumulative survival functions arising from the two mortality processes to assess their contributions by age. Fig 3 shows the intrinsic and extrinsic cumulative survival curves for the twins and the general population. The model fits to the observed cumulative survival data are shown in Fig 4.

**Fig 5:**
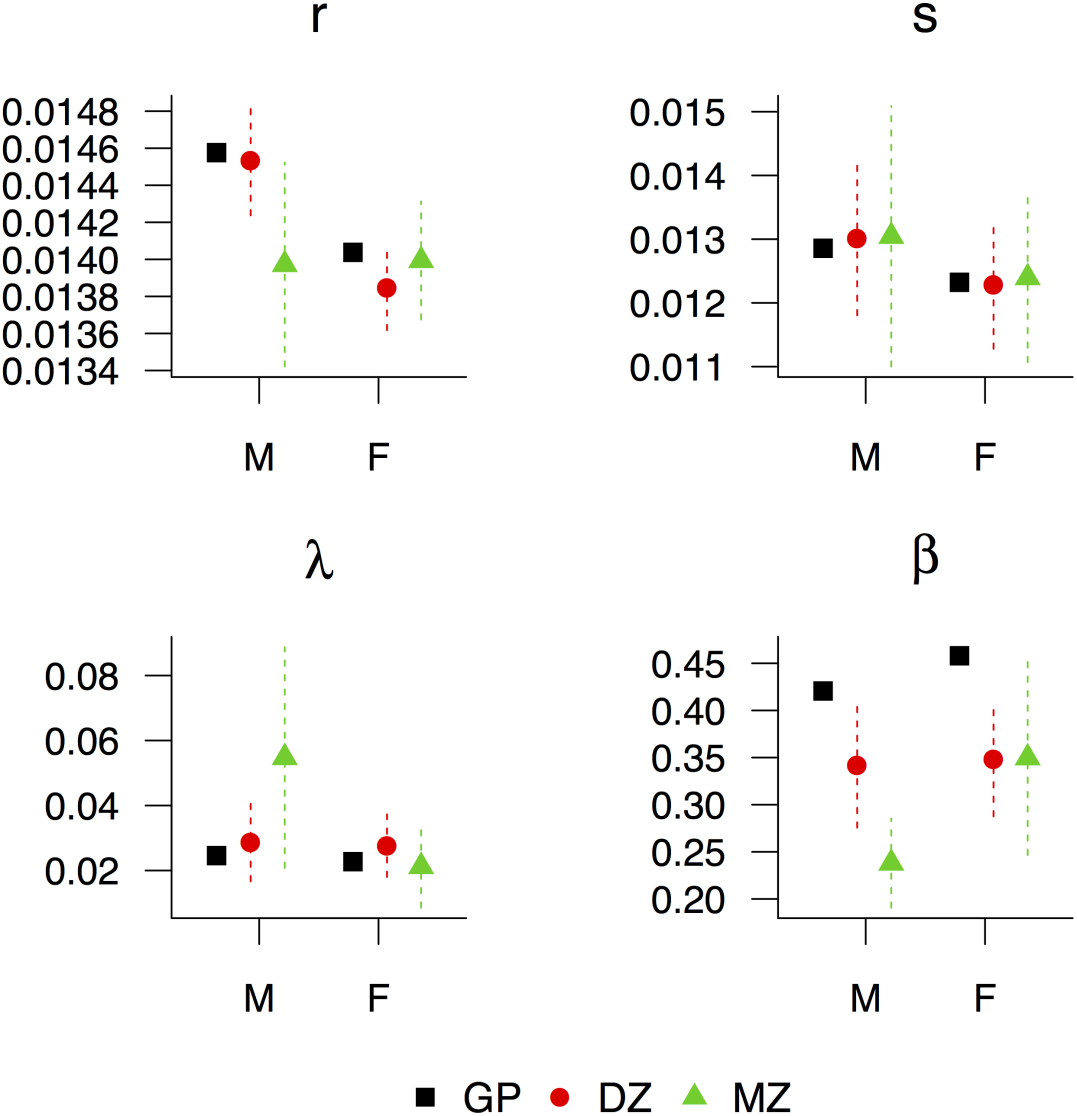
Bias corrected vitality parameters. Bias corrected vitality parameters by zygosity and sex for the 1870–1900 Danish twin cohorts and 1870–1900 Danish general population cohort. The vertical, dashed lines represent uncertainty intervals as +/- 2*standard error. GP: general population, DZ: dizygotic, MZ: monozygotic, M: male, F: female.

For females, there is virtually no difference in intrinsic survival between either zygosity or the general female population (solid lines in Fig 3a). The difference in age-specific survival among females lies with extrinsic mortality where MZ females have a survival advantage over DZ females and the general population across the entire life course (dashed lines in Fig 3a). Similarly, DZ females have an extrinsic survival advantage over the general population until roughly age 90. The bias-corrected fitted model parameters, which are shown in Fig 5, can lend some additional detail to the fitted survival curves (In the interests of accepted scientific practice and for the reader’s benefit this figure also shows the confidence intervals as two standard errors—calculated by taking the square root of the corresponding elements of the estimated variance matrix [30]—on either side of the parameter estimate. However, we caution against over interpreting these intervals since the size of the standard error depends on the initial cohort size, which is obviously much larger for the general population than for either twin group, yielding seemingly large errors for the twin groups by comparison.). Among females there is of course very little difference in the intrinsic parameters, but MZ females do have a slightly lower frequency of extrinsic challenges (parameter λ) compared to DZ females and both female zygositites experience lower challenge magnitudes (parameter β) compared to the general population leading to lower extrinsic mortality rates and greater survival at nearly every age.

While MZ males also see an advantage in extrinsic mortality for most of life, this relationship reverses at about age 70 (dashed lines in Fig 3b). Unlike female twins where the entire survival advantage can be attributed to lower extrinsic mortality, MZ males have lower intrinsic mortality rates at ages >65 indicating the survival advantage for MZ males is driven by lower extrinsic mortality in midlife and lower intrinsic mortality in old age compared to DZ males and the general population. Again, these patterns are detailed in the model parameter estimates (Fig 5). The MZ male intrinsic survival advantage is driven by a smaller *r*, the rate of vitality loss, since, like females, *s* is similar for the groups we compare here. While MZ males do have a greater frequency of challenges, which taken by itself might indicate higher extrinsic mortality, note from Eq 3 that extrinsic survival is dependent on not just the frequency and magnitude of extrinsic challenges but also the rate of vitality loss. The smaller *r* indicates a slower rate of vitality loss for MZ males giving them higher vitality paths overall, which makes them less susceptible to high magnitude challenges relative to DZ males and the general population. Not only are MZ males less susceptible to environmental challenges, they also experience smaller magnitude challenges as indicated by the smaller β. Likewise, DZ males have smaller magnitude challenges compared to the general population. In sum, the MZ male survival advantage is driven by lower extrinsic mortality earlier in life marked by smaller magnitude environmental challenges and lower intrinsic mortality later in life marked by a slower rate of vitality loss, while the DZ male survival advantage over the general population is driven primarily by smaller magnitude challenges.

The survival advantage of MZ males over DZ males at advanced age can be explained by the slower rate of vitality loss for MZ males, but bear in mind that differences in *r* reflect not only genetic differences but also differences in cumulative health behaviors over the life course (e.g. smoking, diet). In terms of vitality loss, it is unlikely that MZ twins as a group carry a special genetic predisposition for longer lives compared to DZ twins or the general population. A plausible alternative explanation for these survival advantages at old age is that the social closeness of MZ twins over DZ twins may encourage positive cumulative health behaviors throughout life (e.g. avoidance of smoking) that translate to higher survival proportions at advanced age for MZ males.

For both sexes survival advantages in midlife by zygosity are explained by differential characteristics of extrinsic challenges. Extrinsic challenges can include any number of direct causes of death, e.g. intentional violence, accidents, acute illness, etc., so the smaller frequency and magnitude of challenges leading to greater survival among twins indicates a protective effect against extrinsic challenges that lead to death.

Both the protective effect against intrinsic death for MZ males at high ages and the protective effect against extrinsic death for both sexes at younger ages are akin to a marriage protection effect. A marriage protection refers to the fact that married adults are generally healthier and at lower mortality risk than unmarried adults [31–34]. It is hypothesized that marriage is associated with good health and lower mortality risk because marriage itself has some beneficial aspect or that having a partner can encourage various good health practices [35], but it may also be that healthier people select into marriage [36] rendering the association between marriage and good health spurious. The study at hand documents a similar association between zygosity and survival; however, twins do not have the confounding issue of self-selection into the lower mortality risk group.

Previous studies also suggest marriage protection effects tend to benefit male partners more than female ones [31, 34, 37, 38], which could explain why the rate of vitality loss is lower for MZ males compared to DZ males, but not lower for MZ females compared to DZ females. Furthermore, the difference in extrinsic challenge magnitude between MZ and DZ twins is larger among male twins than female twins (Fig 5) and Li and Anderson [18] show that over two centuries of historical Swedish mortality data, challenge frequency is about 20% less for females reflecting known risk-taking differences by sex. The sex differential in the model parameters indicates the ‘twin protection effect’ mirrors marriage protection effects in that male twins stand to benefit more from these extrinsic protective effects than female twins.

### Validation

Finally, while we have high quality cohort data on the general population and all twins born in Denmark at the end of the 19^th^ century and assume any differences in survival among these groups are due to zygosity or twin relationship, the twin data are few relative to the national sample and may not be representative of mortality between twin groups or the general population in other countries or time periods. Because the twin data set is relatively small compared to the size of general population data, some unobserved mortality condition may result in an unusual survival pattern in these twin cohorts, which our parameter estimates would reflect.

**Fig 6:**
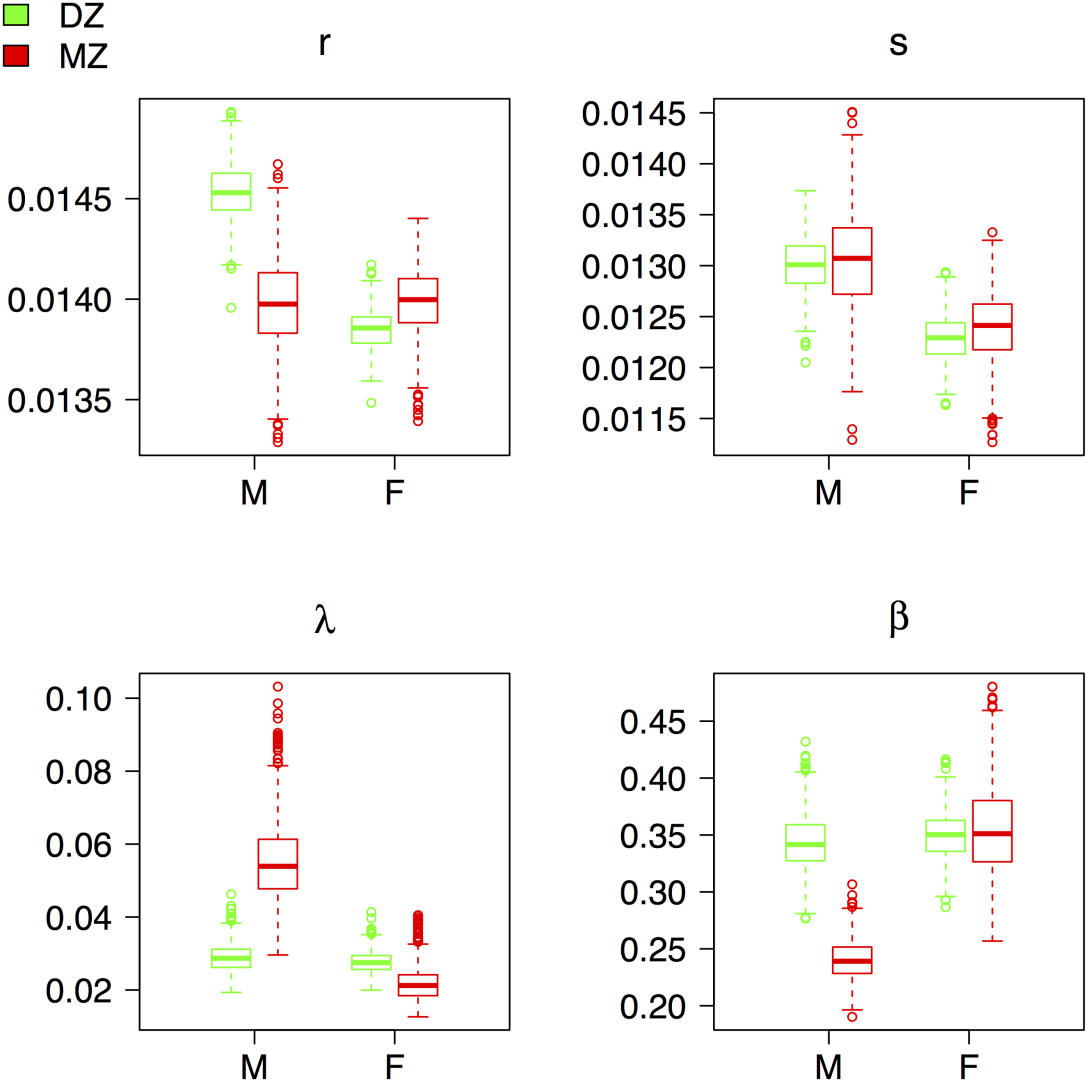
In-sample validation results. Each boxplot shows the distribution of bias-corrected parameter values after iteratively fitting a random 75% sample of twin pairs by sex and zygosity 1,000 times. MZ twins in red and DZ twins in green. Circles outside the ‘whiskers’ of boxplot in this figure represent extreme or outlying estimates for a given iteration. DZ: dizygotic, MZ: monozygotic.

To address this issue, we performed a validation exercise in which the model was fit to randomly selected subsets (75%) of the twin data for each zygosity. Repeating this process 1,000 times yields a distribution of model parameters that were compared to the parameter estimates derived from the full data set. The bias-corrected parameter distributions are presented in Fig 6 and largely confirm the full sample fitting. Females show almost no difference in intrinsic parameters with almost the full female advantage arising from extrinsic mortality. Male MZ twins also have an extrinsic advantage but coupled with a slower rate of vitality loss leading to an intrinsic survival advantage at advanced ages.

## Discussion

We fit a two-process vitality model by sex and zygosity to cumulative survival data from the Danish Twin Registry and by sex to data from the Human Mortality Database for the 1870–1900 Danish general population cohort to investigate the age-dependent effect of acute, extrinsic mortality and chronic, intrinsic mortality processes on survivorship patterns. Because there is often a singular focus on the force of mortality by age, little research has partitioned total mortality into these underlying processes. The vitality framework allows for the decomposition of age-specific survival patterns into extrinsic and intrinsic components and therefor the ability to isolate protective effects at different parts of the age range. When combined with the research advantages of twin data, the two-process model serves as a unique means of assessing the impact of zygosity and social and environmental factor interactions on human survival.

Overall, we find a survival advantage for MZ twins over DZ twins of both sexes at nearly every age and of DZ twins over the general population, but that different processes confer these advantages at different ages. For females, the survival advantage at all ages can be attributed to lower extrinsic mortality rates. Among males, extrinsic advantages account for the survival advantage up to about age 65 where the overall survival advantage begins to narrow and MZ males show better intrinsic survival than DZ males and DZ males show better intrinsic survival compared to the general population.

This research has documented a ‘twin protection effect’ akin to a marriage protection effect where a socially close relationship contributes to better survival outcomes throughout most of life. Notably, while we find evidence for a health protection effect arising from zygosity, the use of twin data allows us to avoid the confounding issue of self-selection that studies of marriage and health often encounter. Research on marriage protection effects as well as the findings presented in this paper are part of a larger body of literature that documents the importance of social support and cohesion for mortality and longevity outcomes [39–41]. In this case greater survival for MZ twins over DZ twins and DZ twins over the general population is driven by lower extrinsic mortality at most ages, which is a likely consequence of the social bond between twins buffering against risky behaviors, providing emotional or material assistance during times of stress exposure, and promoting health-enhancing behaviors [39, 40].

We do not have further information on cause of death, marital status, or social circumstances among this set of twins that would allow further inquiry into the mechanism behind the extrinsic survival advantage at younger ages, but further research in this area may lead to policy recommendations on improving social support at younger ages to lower mortality risk especially among young adult and working-age males.

## Supporting Information

S1 Appendix Two-process vitality model bias correction formulas. (PDF)
