# Correction: A Twin Protection Effect? Explaining Twin Survival Advantages with a Two-Process Mortality Model

**DOI:** 10.1371/journal.pone.0171794

**Published:** 2017-02-02

**Authors:** David J. Sharrow, James J. Anderson

In the Methods section, the third equation in the section titled "Estimating Model Parameters" is incorrect. Please view the complete, correct equation here:

$$l_{x}^{e}=\exp\left(-\frac{\lambda \beta }{r}\exp\left(-1/\beta \right)\left(\exp\left(rx/\beta \right)-1\right)\right)$$
