# Supplementary material for: A Twin Protection Effect? Explaining Twin Survival Advantages with a Two-Process Mortality Model
Source: PLoS One. 2016 May 18;11(5):e0154774. doi: 10.1371/journal.pone.0154774 (PMC4871430; doi:10.1371/journal.pone.0154774)
Supplement: S1 Appendix — (PDF) [file pone.0154774.s001.pdf]

S1 Appendix. Two-process vitality model bias correction formulas.

The following are the bias correction formulas from Li and Anderson [5:350] where the prime symbol (′) indicates the original biased estimate. As Li and Anderson point out the two-process theory cannot be expressed in a closed form solution, but by assuming independent intrinsic and extrinsic processes and that vitality in the extrinsic process is linear and deterministic, they derive an approximate analytical solution that can be fit to data yielding parameter estimates. However, the closed form solution does introduce some bias in parameter estimation. Li and Anderson developed bias correction formulas by first fitting the analytical model to adult mortality curves with a maximum likelihood fitting routine [29]. These are referred to as the biased parameters. They then simulate survival curves from the numerical model using a range of "true parameters" centered over the biased parameters, fit the simulated mortality data with the analytical model, and then develop regressions to relate the "true" parameters with the biased ones.

$$r \approx \frac{r'}{1.06 - 7.26s'} \qquad s \approx \frac{s'}{0.81 + 19.46s'} \qquad \lambda \approx \frac{\lambda'}{0.83 + 19.94s'} \qquad \beta \approx \frac{\beta'}{1.02 - 3.38s'}$$

See Li, Ting and James J. Anderson. 2013. "Shaping Human Mortality Patterns Through Intrinsic and Extrinsic Vitality Processes." Demographic Research 28: 341-372 for further details on model development and bias correction.
